# Data from the ACHILLES (Assessing Daily Changes in Individual Prerequisites of Learning Success) Project

**DOI:** 10.5334/jopd.182

**Published:** 2026-10-15

**Authors:** Jasmin Breitwieser, Garvin Brod

**Affiliations:** 1 DIPF | Leibniz Institute for Research and Information in Education, DE; 2 Leibniz-Institut für Wissensmedien, DE

**Keywords:** self-regulated learning, high-stakes exam, process data, learning diary, log data

## Abstract

This dataset contains intensive longitudinal data on self-regulated learning processes and learning performance among German medical students preparing for the second written state examination in 2018. Data were collected online and include repeated questionnaires and working memory assessments, as well as objective indicators of learning behavior on a digital learning platform. In addition, the study involved an intervention, with two-thirds of students receiving planning prompts. The dataset includes pre-, post-, and daily ambulatory assessments as well as log data-based indicators of learning behavior from up to 365 participants. It is provided in non-proprietary formats with extensive documentation that facilitates reuse.

## (1) Background

Self-regulated learning (SRL) is a key competence that enables students to learn effectively, adapt their learning strategies to changing task demands, stay motivated, and persist when facing challenges. Inter-individual differences in various aspects of SRL predict academic performance in both traditional learning settings (Dent & Koenka, 2016; Richardson et al., 2012) and in digital learning environments (Broadbent & Poon, 2015; Cheng et al., 2023). Consequently, SRL has been a major focus of educational research for several decades (Tinajero et al., 2024; Winne, 2005).

The ACHILLES (Assessing Daily Changes in Individual Prerequisites of Learning Success) project is grounded in the conceptualization of SRL as involving cognitive, metacognitive, and motivational-emotional aspects of learning that are interrelated through a three-phase process (Schmitz & Wiese, 2006; Zimmerman, 2000). First, prior to studying, students set goals and strategically plan their approach to the task. In addition, motivational beliefs and affective states influence how students engage with the task. Second, during studying, students use self-control and self-observation to optimize their efforts, apply appropriate learning strategies, and monitor their progress. Third, after studying, students evaluate their performance in relation to their goal and derive conclusions for future adjustments to their learning approach. The outcomes of this evaluation are expected to inform subsequent self-regulation of learning. Taken together, this perspective conceptualizes SRL as a cyclical, intra-individual process that unfolds over time and over multiple study sessions.

A major contribution of the ACHILLES project lies in the provision of rich, multimodal data capturing cognitive, metacognitive, and motivational-emotional SRL processes in the context of medical students preparing for the second state examination in Germany. Preparing for this high-stakes exam involves an intensive period of daily, self-directed studying lasting several months. Students in this population use the online learning platform AMBOSS (https://www.amboss.com) to prepare for the exam. This provides a unique opportunity to investigate SRL processes in an authentic digital learning environment.

With regard to cognitive processes, ACHILLES draws on research on effective learning strategies. Preparation for the state examination is largely based on revisiting and consolidating previously acquired knowledge. The primary learning activity, which the learning platform AMBOSS aims to support, is practice testing with complex multiple-choice questions. When distributed across multiple study sessions, practice testing is considered one of the most effective learning strategies (Bjork et al., 2013). A second central activity on the digital learning platform involves studying flashcards that summarize exam-relevant content. These activities provide a structured context in which the use of cognitive learning strategies can be captured through both self-reports and behavioral trace data.

In addition to learning strategy use, students’ capacity to self-regulate their learning may also depend on more basic cognitive resources available to them. Cognitive functions such as working memory are assumed to enable higher-order self-regulation and metacognition (Theobald et al., 2025). Empirical evidence suggests that working memory performance fluctuates from day to day and that these fluctuations are associated with factors such as affect and sleep (Brose et al., 2014; Könen et al., 2015).

With regard to metacognitive processes, students’ self-evaluations after learning are a central metacognitive strategy (Ku & Ho, 2010). After learning, self-regulated students should judge their performance, evaluate whether they achieved their goal, and derive conclusions about how to adjust their approach (Zimmerman, 2000). Metacognitive self-evaluation thus represents a key process through which one learning phase affects the next.

Regarding motivation, theoretical perspectives emphasize the role of value- and expectation-related appraisals (Eccles & Wigfield, 2002; Pintrich, 1999) as well as students’ belief about their capacity to self-regulate their learning and cope with obstacles (i.e., self-efficacy; Bandura, 1997). These appraisals and beliefs underly goal setting and strategic planning, which are key forethought processes in SRL (Zimmerman, 2000).

In addition to students’ goals and motivational appraisals, volitional control can also be considered part of the motivational component of SRL, as it helps individuals translate intentions into action (Koole & Zeng, 2026). Specifically, students may monitor conflicts during learning and regulate themselves to overcome obstacles and disruptions in the pursuit of their learning goals (Corno & Kanfer, 1993; Kuhl, 1984).

Regarding learning-related emotions, Pekrun’s (2006) control-value theory of achievement emotions posits that students experience discrete, outcome- and activity-related emotions in learning contexts (e.g., enjoyment, pride, anger, frustration, boredom) that can facilitate or hinder SRL processes. Relatedly, emotion regulation may play an important role in shaping how these processes unfold (Gross, 2002).

In addition to examining the relationships between cognitive, metacognitive, and motivational-emotional aspects of SRL, the second major aim of the ACHILLES project was to test an intervention designed to improve students’ volitional control while studying. Specifically, the intervention aimed at preventing premature abandonment of study intentions. The intervention was based on the well-established self-regulation strategy of using implementation intentions, also called if-then plans, which have been shown to promote behavior change and prevent goal derailment (Gollwitzer, 1999; Gollwitzer & Sheeran, 2006). Implementation intentions specify a link between a relevant situational cue and a goal-directed action, typically in the form of: “If/when situation X occurs, then I will perform behavior Y.” In the present study, prompting students to internalize an if-then plan had positive effects on daily goal achievement and exam performance (Breitwieser et al., 2022).

## (2) Methods

### 2.1 Study design

Data collection comprised four phases: 1) a one-time pre-measure administered prior to the ambulatory assessment period, approximately 9-10 weeks before the medical students took the exam; 2) an ambulatory assessment period involving daily self-reports and a working memory task, repeated for up to 40 days; 3) a one-time post-measure after the ambulatory assessment period; and 4) students’ self-report of their exam performance. Figure 1 provides an overview of the data collection procedure. All self-report data were collected using Research Electronic Data Capture (REDCap; Harris et al., 2009). The working memory task was programmed with PsychoPy (Peirce et al., 2019) and hosted on an on-premises server.

**Figure 1:**
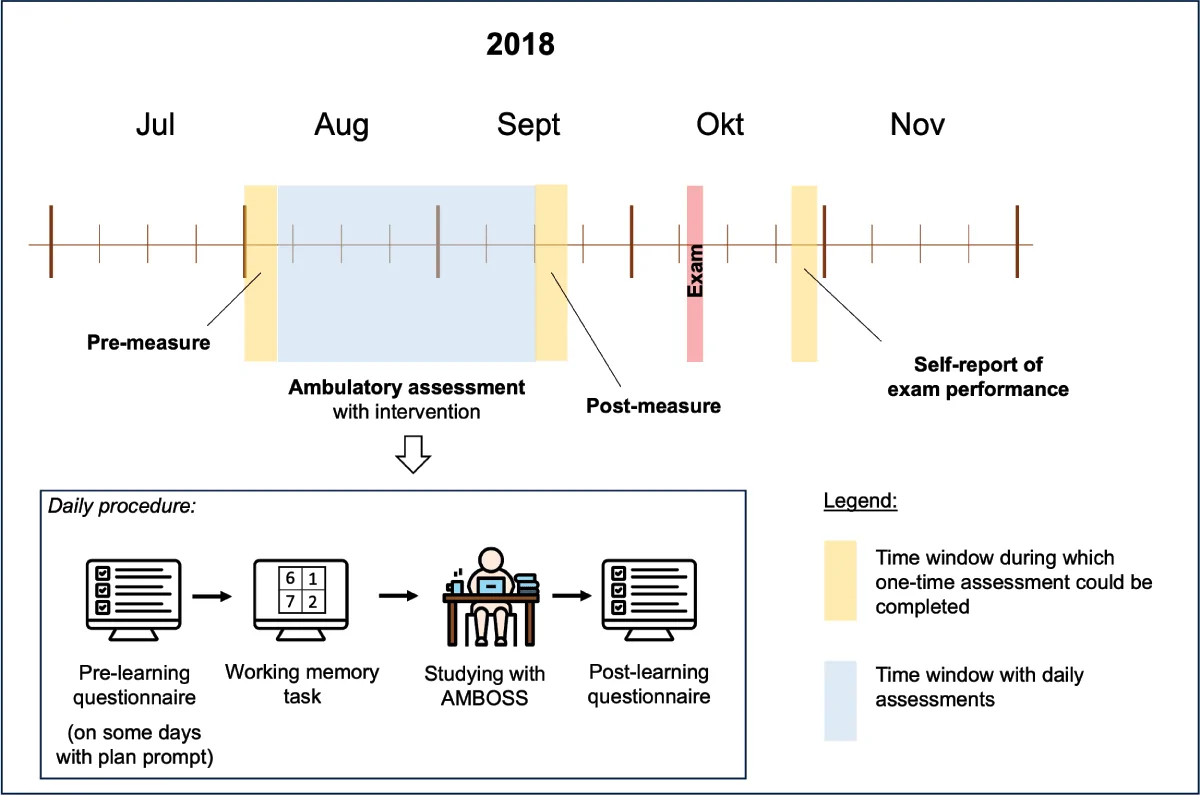
Overview of Data Collection. *Note*. The pre-measure, post-measure, and self-report of exam performance were administered once. However, students had a time window of several days to complete these questionnaires. For an overview of the days on which the plan prompt was administered and of the working memory task procedure, see Figures 2 and 3, respectively. © Breitwieser & Brod (2026). Reproduced with permission.

The pre-measure involved a series of self-report questionnaires about demographics and learning-related constructs (e.g., study motivation; see Table 2 for an overview of all constructs). After the questionnaires, students completed the web-based working memory task. Data from the pre-measure enable the investigation of inter-individual differences in the assessed constructs.

The ambulatory assessment period began after completion of the pre-measure and was intended to last 30 days. Due to an incorrect setting in REDCap, however, questionnaires were administered for 40 days. While some students discontinued participation after 30 days (the communicated study duration), others continued for up to 40 days. During this period, students completed brief questionnaires each day both before and after studying. At the end of the pre-study questionnaire, students were directed to a web-based working memory task, which they were asked to complete daily. These intensive longitudinal data allow for within-person analyses, such as examining temporal trends across the study period and intra-individual associations between learning-related constructs. In addition, the data can be used to investigate inter-individual differences, for example by aggregating construct scores across time points for each student and comparing these averages between individuals.

A planning intervention aimed at supporting students in achieving their daily learning goals was implemented during the ambulatory assessment period. After completion of the pre-measure, students were randomized in a 2:1 ratio to either the intervention group (n = 240) or the control group (n = 119). On half of the study days of the ambulatory assessment period, participants in the intervention group received a prompt to internalize a pre-specified if–then plan. This prompt was delivered as an additional page within the pre-study questionnaire, presenting the plan and asking students to internalize it. On the remaining days in the intervention group, and on all days in the control group, this page was not shown. A detailed description of the intervention, including an evaluation of its effectiveness, is provided in Breitwieser et al. (2022) and in the accompanying methodology report of the dataset (Breitwieser & Brod, 2026).

For the post-measure, students were again given several days to complete the assessment. Similar to the pre-measure, it consisted of a series of self-report questionnaires on learning-related constructs, many of which were identical to those used in the pre-measure. Combining data from the pre- and post-measures enables the investigation of changes in these constructs over the 30- to 40-day period between assessments.

Approximately two weeks after the exam, students were asked to report their exam results.

In addition to the four data collection phases, the ACHILLES dataset includes log data-based indicators of students’ behavior on the digital learning platform AMBOSS, which students used to prepare for the exam.

### 2.2 Time of data collection

Table 1 provides an overview of the data collections, including the time periods during which they took place.

**Table 1:** Overview of Data Collections.


DATA COLLECTION	ASSESSMENT TYPE	TIME	TYPE OF DATA	*N*	NUMBER OF VARIABLES

Pre-measure	One-time	30.07.-07.08.2018	Demographic	365	3

			Questionnaires	365	104

			Working memory task	351	20

Ambulatory assessment	Daily for up to 40 days	06.08.-17.09.2018	Pre-learning questionnaire	359	46

			Post-learning questionnaire	352	45

			Working memory task	355	16

Post-measure	One-time	15.09.-21.09.2018	Questionnaires	330	65

Exam performance	One-time	About^a^ two weeks after the exam^b^	Self-report	343	2

Data from the digital learning platform (AMBOSS)	Daily for up to 179 days	13.04.-08.10.2018	Behavioral indicators based on log data	361	23


^a^ The exact time stamps when the data collection took place have not been preserved. ^b^ The exam took place from 9–11 October 2018.

### 2.3 Location of data collection

The ACHILLES data were collected from medical students in Germany, with all data collection conducted online.

### 2.4 Sampling, sample and data collection

The target population was medical students. Starting in July 2018, medical students in Germany were recruited who planned to take the second written state examination in the fall of 2018 and who used the AMBOSS learning platform as their primary resource for preparation. The students were recruited nationwide with the support of AMBOSS through newsletters and social media channels, as well as through Facebook groups for medical students. The study therefore relied on convenience sampling.

In total, 365 participants contributed data (*mean age* = 26.17, *SD* = 3.79, range 22–53 years; 70.14% female). Table 1 provides an overview of participation across the different study phases. As is typical for ambulatory assessment studies, completion rates varied across study days, resulting in missing observations at the within-person level. That being said, the participation rate was quite high, with each participant completing an average of 29.40 (*SD* = 7.73) pre-learning questionnaires and 28.29 (*SD* = 6.91) post-learning questionnaires.

Participants received financial compensation for participation. They received €5 each for completing the pre- and post-measure assessments. In addition, participants received €1 for each day of the ambulatory assessment data collection period with complete data (questionnaires before and after studying) and a bonus of €10 if they provided complete data for at least 30 days. The maximum possible compensation was therefore €50.

### 2.5 Materials/ Survey instruments

A comprehensive description of all instruments and study materials, including questionnaire items, instructions, and task descriptions, is provided in the accompanying Methodology Report, which is available on the website in both German and English (https://doi.org/10.7477/1242-435-0). The report includes the exact wording of all self-report items, response scales, participant instructions, information on questionnaire timing, and screenshots of the survey implementation. In addition, it contains detailed descriptions of the working memory task, the intervention, and the digital learning platform AMBOSS, including illustrative screenshots.

#### 2.5.1 Self-Report Questionnaires

Table 2 provides an overview of the scales and constructs assessed through self-report questionnaires.

**Table 2:** Overview of self-report measures administered during the study.


DATA COLLECTION	CONSTRUCT	SCALE	NUMBER OF ITEMS

Pre-measure	Multitasking	Self-created	8

	Exam goal	Self-created	1

	Motivation regarding exam goal Importance Expectancy Commitment	Self-created	3

	Emotions Enjoyment Anxiety	Achievement Emotions Questionnaire (Pekrun etal., 2002)	10

	Motivation Intrinsic goal orientation Extrinsic goal orientation Control of learning beliefs Expectancy for success Task value	Motivated Strategies for Learning Questionnaire (Pintrich & De Groot, 2012)	15

	Self-efficacy for self-regulated learning	Self-created based on the Self-Efficacy for Self-Regulated Learning scale (Bandura, 2006)	4

	Self-regulated learning Goal Setting Planning Monitoring Regulation Procrastination	Self-created, partly based on the “Lernstrategien im Studium” questionnaire (Wild & Schiefele, 1994), the self-regulated learning questionnaire by Dörrenbächer and Perels (2016), and the procrastination scale by Tuckman (1991)	18

	Stress	Perceived Stress Scale (Klein et al., 2016)	10

	Emotion regulation Reappraisal Emotional expression Emotional experience	Heidelberg Form for Emotion Regulation Strategies (Izadpanah et al., 2019)	12

	Self-control	Brief Self-Control Scale (Sproesser et al., 2011)	13

Ambulatory assessment – pre-learning questionnaire	Interest	Self-created	1

	Daily goals for studying on the digital learning platform Number of questions to practice Number of flashcards to read	Self-created	2

	Motivation regarding daily goals Importance Expectancy Commitment	Self-created	6

	Control beliefs / self-efficacy	Self-created based on control-value theory (Pekrun, 2006)	4

	Sleep Length Quality	Self-created	2

	Emotions Anxiety Anger Boredom Enjoyment	Adapted from Achievement Emotions Questionnaire (Pekrun et al., 2002)	4

Ambulatory assessment – post-learning questionnaire	Metacognitive self-evaluation	Self-created	3

	Multitasking	Self-created	1

	Cognitive learning strategies used while reading flashcards	Self-created	7

	Feeling (over-/under-) challenged while learning	Self-created	1

	Emotions during learning: Anxiety Anger Boredom Enjoyment	Adapted from Achievement Emotions Questionnaire (Pekrun et al., 2002)	4

	Emotions after learning: Anxiety Anger Pride Enjoyment	Adapted from Achievement Emotions Questionnaire (Pekrun et al., 2002)	4

	Volitional control Monitoring Regulation Procrastination	Self-created	9

	Stress anticipation	Items from Kramer et al. (2019)	3

Post-measure	Same items as in pre-measure: Emotions Motivation Self-efficacy for self-regulated learning Self-regulated learning Stress		42

	Learning Strategies Rehearsal Organization Elaboration	Adapted from “Lernstrategien im Studium” questionnaire (Wild & Schiefele, 1994)	12

	Beliefs about the planning intervention	Self-created	6

	Measurement reactivity	Self-created	2


#### 2.5.2 Working memory task

The working memory task was based on the paradigm introduced by Riediger and colleagues (2011). Each trial consisted of a 2×2 grid containing four digits ranging from 0 to 9 (see Figure 2 for a schematic overview of a trial sequence). The initial grid was displayed for 6,000 ms. After an interstimulus interval (ISI) of 250 ms, five updating operations were presented sequentially within the cells of the grid. The updating operations consisted of additions and subtractions ranging from –8 to +8. All intermediate and final results remained within the range of 0 to 9. After presentation of all five operations, participants were instructed to enter via keyboard the final value for each of the four cells. Responses could not be corrected once entered. After completing each trial, participants received feedback indicating the number of correct responses (e.g., “3 out of 4 numbers correct”).

**Figure 2:**
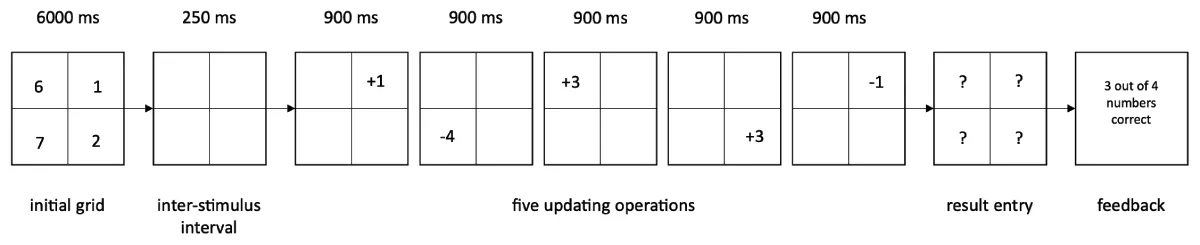
Schematic Overview of the Working Memory Task Trial Sequence. *Note*. © Breitwieser & Brod (2026). Reproduced with permission.

The updating operations were presented for 900 ms during the main task blocks. In the pre-measure, participants first completed two practice blocks to familiarize themselves with the task. The first practice block consisted of three trials with updating operations presented for 2,700 ms each. The second practice block also consisted of three trials, with a presentation duration of 1,800 ms. Participants then completed the main task block, which comprised 15 trials with a presentation duration of 900 ms. The full task required approximately 10 minutes to complete during the pre-measure. During the ambulatory assessment period, participants completed a shortened version of the working memory task each day consisting of six trials, requiring approximately 3 minutes to complete. The set of trials varied across study days; however, all participants received the same trial set on a given day.

#### 2.5.3 Experimental design

The experimental design included one between-person factor and one within-person factor. The between-person factor was group assignment (intervention group vs. control group; variable name: group). The within-person factor was the presence versus absence of the intervention prompt on a given study day (variable name: encouragement). The days on which the intervention prompt appeared were predetermined. Two to three days with an intervention prompt were alternated with two to three days without an intervention prompt (Figure 3). This design was chosen to investigate how the effectiveness of the prompts changes over consecutive days with vs. without a prompt (see Breitwieser et al., 2022). To control for order effects, the intervention group was divided into two subgroups that had two different sequences of days with and without prompts.

**Figure 3:**
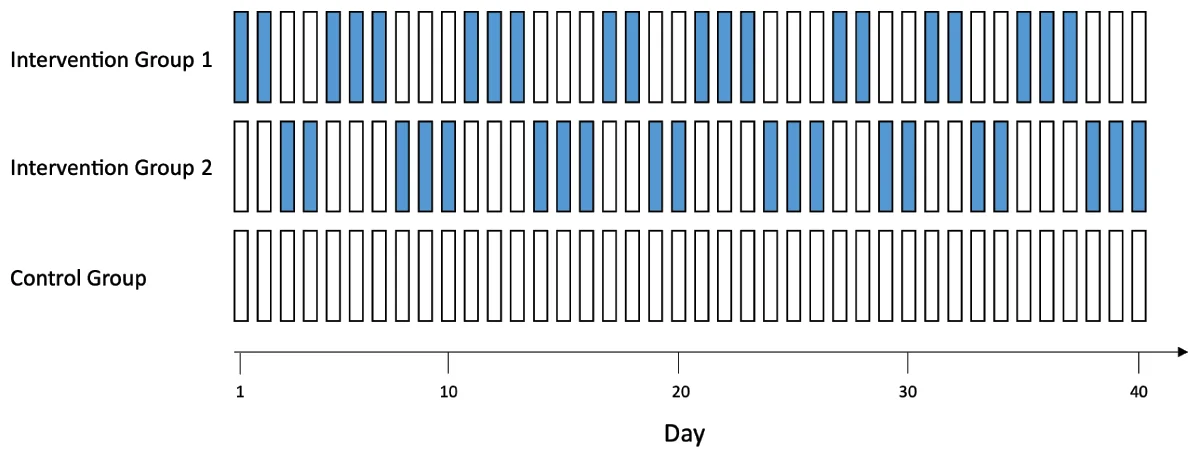
Schematic Overview of the Intervention Procedure in All Groups. *Note*. Days with an intervention prompt are shown in blue. © Breitwieser & Brod (2026). Reproduced with permission.

#### 2.5.4 Digital learning platform AMBOSS

AMBOSS (https://www.amboss.com) is a digital learning platform for medical students and physicians that has been widely used in Germany since its launch in 2012. According to the platform provider, more than 95% of students use AMBOSS to prepare for the second written state examination. The platform is available both as a web application and as a mobile app.

AMBOSS provides an extensive library of multimedia learning materials (“flashcards”) covering the full medical curriculum, as well as thousands of multiple-choice questions from previous state examinations. In addition, the platform offers a structured 100-day study plan for exam preparation. This plan distributes relevant learning materials and exam questions across 100 days, while intentionally mixing question topics to avoid simple item recognition based on the day’s assigned content. During the ambulatory assessment period, participants were at different stages of this study plan. In the daily pre-learning questionnaire, participants reported up to three topics they intended to study that day.

The Methodology Report accompanying the dataset includes screenshots of the platform. However, because AMBOSS is a commercial platform, comprehensive documentation of its proprietary materials cannot be provided. Furthermore, contractual agreements with the platform provider prohibit sharing the original platform log data. Instead, the dataset includes 20 derived indicator variables describing students’ learning behavior on AMBOSS between April 13, 2018 (the day after the spring 2018 exam), and October 8, 2018 (the day before the fall 2018 exam). These indicators capture several aspects of students’ daily learning behavior on AMBOSS. For each student and day, the dataset includes the number of questions answered, the number of flashcards accessed, the number of correctly answered questions, and the timestamps of the first and last question and flashcard activity. Additional variables distinguish between all activities and unique activities (i.e., counting each question or flashcard only once when repetitions occurred). Furthermore, the indicators are available for two different definitions of a “day”: a conventional calendar day (midnight to midnight) and a shifted day definition (5:00 a.m. to 5:00 a.m.) to account for students who continued studying beyond midnight. The shifted indicators were used in previous analyses because they better account for study sessions extending beyond midnight; the non-shifted indicators are provided for documentation purposes. An overview of all 20 indicators is provided in Table 3.

**Table 3:** Overview of Indicator Variables Derived From AMBOSS Log Data.


VARIABLES	DESCRIPTION

n_qs n_qs_shifted	Number of questions answered per day

n_unique_qs n_unique_qs_shifted	Number of unique questions answered per day

n_qs_correct n_qs_correct_shifted	Number of questions answered correctly per day

n_unique_qs_correct n_unique_qs_correct_shifted	Number of unique questions answered correctly per day

n_lcs n_lcs_shifted	Number of flashcards clicked per day

n_unique_lcs n_unique_lcs_shifted	Number of unique flashcards clicked per day

start_time_qs start_time_qs_shifted	Timestamp of the first question answered per day

end_time_qs end_time_qs_shifted	Timestamp of the last question answered per day

start_time_lcs start_time_lcs_shifted	Timestamp of the first flashcard clicked per day

end_time_lcs end_time_lcs_shifted	Timestamp of the last flashcard clicked per day


### 2.6 Quality Control

Several measures were implemented to ensure data quality. Missing data were coded using a standardized scheme that distinguishes between different reasons for missingness. For example, separate codes indicate values missing due to questionnaire filter logic, missing by design, or questionnaires not completed on a given day. The coding scheme for missing values is documented in the data dictionaries accompanying the dataset.

In addition, checks were conducted to ensure that all data files could be reliably merged using the common identifiers for participants and study days. Data processing procedures further included checks for completeness, consistency of variable formats, and plausibility of values.

### 2.7 Data anonymisation and ethical issues

Ethics approval was obtained from the ethics committee of DIPF | Leibniz Institute for Research and Information in Education. All participants provided written informed consent prior to participation.

Participants were identified only through pseudonymized participant IDs. No directly identifying personal information was collected as part of the study. In addition, the only free-text field in the dataset in which participants in the intervention group wrote down the if–then plan they were asked to internalize was manually reviewed to ensure that it did not contain personally identifying information before inclusion in the shared dataset.

### 2.8 Existing use of data

To date, the dataset has been used for eight published papers (Berweger et al., 2025; Breitwieser & Brod, 2022; Breitwieser et al., 2019; 2022; Lin et al., 2026; Theobald et al., 2021, 2022, 2023) and one preprint (Nobbe et al., 2025) that is also submitted for publication in a peer-reviewed journal. All studies used variables from the shared dataset, including indicators derived from the log data of the learning platform.

## (3) Dataset description and access

### 3.1 Repository location

The dataset is available at the Research Data Centre for Education (https://www.fdz-bildung.de) with the DOI https://doi.org/10.7477/1242-435-0.

### 3.2 Object/file name

The deposited dataset is provided as a ZIP archive named S1242_Dataset_ACHILLES.zip. The archive contains the data files organized into folders corresponding to the different data collection phases and types of data as listed in Table 1. An overview of all file groups, their formats, and English descriptions of the content is provided in Table 4. The naming convention used for the individual log files of the working memory task is described in the table note.

**Table 4:** Overview of files.


FOLDER/FILE NAME	FILE FORMAT	NO. OF FILES	LEVEL OF OBSERVATION	CONTENT

Prae-Messung/DemographischeDaten	CSV, RDS	2	Participant	Pre-measure demographic data

Prae-Messung/PraeMessung_Fragebogen	CSV, RDS	2	Participant	Pre-measure questionnaire data

Prae-Messung/PraeMessung_Arbeitsgedaechtnisaufgabe	CSV, RDS	2	Participant	Pre-measure working memory task data

Prae-Messung/PraeMessung_Arbeitsgedaechtnisaufgabe_Logfiles	CSV	351	Task	Pre-measure working memory task log files; one file per recorded task session

Ambulatorisches Assessment/AmbulatorischesAssessment_FragebogenVorDemLernen	CSV, RDS	2	Participant × study day	Ambulatory assessment pre-learning questionnaire data

Ambulatorisches Assessment/AmbulatorischesAssessment_FragebogenNachDemLernen	CSV, RDS	2	Participant × study day	Ambulatory assessment post-learning questionnaire data

Ambulatorisches Assessment/AmbulatorischesAssessment_Arbeitsgedaechtnisaufgabe	CSV, RDS	2	Participant × study day	Ambulatory assessment working memory task data

Ambulatorisches Assessment/AA_Arbeitsgedaechtnisaufgabe_Logfiles	CSV	10,402	Task session	Ambulatory assessment working memory task log files**;** one file per recorded task session

Post-Messung/PostMessung	CSV, RDS	2	Participant	Post-measure questionnaire data

Indikatorvariablen_Lernplattform/AMBOSS_Indikatorvariablen	CSV, RDS	2	Participant × study day	Indicator variables derived from the AMBOSS learning platform

Examensergebnis/Examensnote	CSV, RDS	2	Participant	Exam grade


*Note*. All data files except for log files from the working memory task are provided in both .csv and .rds format. The log files are provided as individual .csv files. Log-file names follow the convention [participant ID]_[study day]_[timestamp]_tidy.csv. For example, 454_day15_2018-08-22 08_10_33_tidy.csv refers to participant 454, study day 15, and a recording timestamp of August 22, 2018, at 08:10:33.

Datasets with participant as the level of observation can be merged using the variable *record_id*. This applies to the datasets from the Pre-measurement, Post-measurement, and Exam result phases. To merge the Ambulatory Assessment datasets, the variable *day* can be used in addition to *record_id*. To merge the learning-platform indicator data with the Ambulatory Assessment datasets, the variable *date* can be used in addition to *record_id*.

### 3.3 Data type

All self-report data is made available as cleaned primary data. We have added codes for missing data, distinguishing between different types of missings (e.g., missing by design, missing due to the questionnaire’s filtering logic, missing because questionnaire was not filled out that day).

Data from the working memory task (collected at pre-measure and during the ambulatory assessment period) is made available as cleaned primary data. Since the data files provided by the PsychoPy experimental software are unstructured, we transformed them into a structured format suitable for further processing. In addition, we provide processed data in the form of variables aggregated from the log data indicating performance on the working memory task (e.g., number of error-free trials per block).

For data from the digital learning platform AMBOSS, the dataset contains processed data derived from the log data of AMBOSS. The data are aggregated at the day and person level, thus providing information on how many questions a student answered on AMBOSS on a given day and how many flashcards they clicked on. Due to legal reasons, the primary log data cannot be made available.

### 3.4 Format names and versions

All files are accessible in the non-proprietary .csv format. In addition, all files except for the primary log data from the working memory task are also available as .rds files, which allow for efficient storage and direct use within R (R Core Team, 2024).

### 3.5 Language

The dataset primarily consists of numeric data. Variable names and some categorical value labels in the dataset are provided in German. The accompanying Methodology Report, including the data dictionaries and study documentation, is available in both German and American English. The English Methodology Report provides item and value labels in English.

### 3.6 License

The dataset is provided under the repository’s Terms of Use, which govern access, use, and sharing of the data.

### 3.7 Limits to sharing

The dataset is not fully openly accessible because participants consented to data sharing exclusively for scientific research purposes. Users who wish to use the data must register with the research data center and verify their identity. In addition, they must briefly explain the intended use of the data and sign a data use agreement. The identity verification and review of the applicant’s institutional affiliation and intended research use ensure that access is provided only to appropriately identified researchers for scientific purposes. Researchers based outside Germany may also apply for access.^1^

### 3.8 Publication date

The dataset was published on 17/06/2026.

### 3.9 FAIR data/Codebook

The dataset adheres to the FAIR standards (findable, accessible, interoperable, and reusable; Wilkinson et al., 2016). First, the data are findable as they are assigned a unique and persistent digital object identifier (DOI). The dataset and its accompanying documentation are registered and indexed on the searchable platform of the research data center. In addition, the data are described using rich metadata, including information on the study design, sampling procedure, and mode of data collection.

Second, access to the data is provided through the research data center, where users can obtain the dataset in accordance with the center’s access regulations: Researchers are granted access once they have registered, briefly explained the intended use and signed a data use agreement. The metadata remain openly accessible even if access to the data may be subject to restrictions.

Third, the dataset is interoperable. All files are provided in the non-proprietary .csv format, ensuring compatibility with a wide range of statistical software environments. All data files contain unique identifiers for participants and study days, which enable researchers to merge datasets flexibly according to their analytical needs. Furthermore, the dataset uses harmonized coding schemes for missing values.

Fourth, the data are reusable, as they are accompanied by detailed documentation. The documentation includes information on study context and procedures, sampling, and data processing steps. In addition, data dictionaries in the accompanying documentation comprehensively document all variables, including variable names, data types, item labels, response scales, value ranges, coding of missing values, and the source instruments from which the variables were derived. Together, these features ensure that researchers can interpret and appropriately analyze the data.

## (4) Reuse potential

The ACHILLES dataset offers substantial potential for reuse. The combination of intensive longitudinal self-report data (e.g., achievement emotions; see Table 2), daily assessments of working memory performance, and objective indicators of behavior on a digital learning platform makes this dataset particularly valuable and difficult to replicate. The dataset includes a broad assessment of different aspects of SRL, including emotional, motivational, cognitive, and metacognitive components. This breadth allows researchers to study SRL processes from multiple perspectives and to consider how inter-individual differences may influence intra-individual dynamics.

Regarding intra-individual processes, previous studies based on the dataset have, for example, investigated how achievement emotions influence daily goal attainment and goal revision (Theobald et al., 2021), or how competence and value beliefs, procrastination, study time, study satisfaction, and goal attainment interrelate (Theobald et al., 2023). The dataset offers many additional possibilities for secondary analyses. For example, researchers could examine how daily self-evaluations and stress anticipation after learning affect study motivation and goal setting the following day. This question is directly related to process models of SRL, which assume that different phases of learning (forethought, performance, and self-reflection) influence one another (Zimmerman, 2002). By enabling the investigation of these dynamic within-person processes in an authentic learning context, the dataset could contribute to the refinement of theoretical models by specifying how SRL processes unfold over time.

A particular strength of the dataset is its integration of self-report and objective behavioral data, enabling researchers to examine how subjective experiences relate to students’ actual daily learning behavior and performance on the digital learning platform. For example, several previous manuscripts that utilized this data have incorporated a measure of daily goal achievement, indicating whether students achieved the study goal they had set for themselves in the pre-learning questionnaire (i.e., number of questions they intended to answer). Goal achievement was thus operationalized by comparing self-reported goals with the objective indicator of how many questions were answered on a given day (e.g., Breitwieser et al., 2019; Lin et al., 2026). Other studies have focused on students’ learning performance, operationalized as the proportion of correctly answered questions (e.g., Theobald et al., 2022). In contrast, the flashcard-related indicators (e.g., number of flashcards viewed each day) have not yet been explored. These variables offer opportunities to investigate how digital content engagement relates to motivation, strategy use, or performance.

In addition to intra-individual processes, the dataset enables research on how individual differences influence day-to-day learning behavior, emotions, motivation, or the use of (meta-)cognitive and volitional control strategies. For example, researchers could examine how trait self-control relates to day-to-day use of volitional strategies (Inzlicht & Roberts, 2024).

Taken together, the dataset provides a rich resource for advancing research on SRL in authentic, digital educational contexts. Key strengths of the dataset include its multi-method assessment approach (self-reports, objective behavioral indicators, cognitive performance measures), its intensive longitudinal design, and its high ecological validity.

Beyond answering new research questions, the dataset also offers many opportunities for teaching purposes. Its complex multilevel structure and combination of multiple data modalities make it well suited for use in advanced statistics courses. In particular, the dataset can be used to teach methods for analyzing nested, intensive longitudinal data, including multilevel modeling, lagged analyses, dynamic structural equation modeling, and other time-series approaches. Furthermore, since missing observations are common in intensive longitudinal studies, the dataset provides a realistic context for teaching and evaluating methods for handling missing data, such as multiple imputation or full-information maximum likelihood estimation.

When using the dataset, several limitations should be considered. First, many questionnaire items were adapted or self-created specifically for the study, particularly for the ambulatory assessment questionnaires. In addition, some constructs (e.g., multitasking, procrastination), were assessed using single-item measures to reduce participant burden during the daily assessments. As a result, psychometric properties may be less well established than for standardized, multi-item scales. However, previous research has suggested that single-item measures can be appropriate when assessing constructs that are narrow, clearly defined, and unidimensional (Allen et al., 2022).

Second, regarding the measurement of working memory performance, it should be noted that the task was administered online in unsupervised settings, meaning that variations in testing environments could not be controlled. Performance increased significantly across the ambulatory assessment period, most likely reflecting practice effects. While the between-person reliability was excellent (ω = .99), the within-person reliability was low (ω = .44), which should be considered when modelling intra-individual fluctuations in working memory performance.

Third, the dataset does not include the raw log data obtained from the digital learning platform. Instead, the dataset contains derived indicator variables summarizing learning behavior. Certain forms of learning analytics, such as sequence-mining approaches, are thus not possible.

Fourth, researchers reusing the data should consider the intervention design when conducting secondary analyses. During the ambulatory assessment period, two-thirds of participants in the intervention group received planning prompts aimed at improving their volitional control (persisting until the learning goal was reached). Therefore, it should be statistically controlled for intervention exposure, or sensitivity analyses should be conducted to examine whether the findings are replicated when the analysis is restricted to the control group.

Finally, the dataset is based on a convenience sample of German medical students preparing for a specific high-stakes examination. Only limited demographic information was collected, which constrains analyses of socio-demographic differences and limits the generalizability of findings to other populations and educational contexts.

## Footnotes

If an applicant’s identity document is not accepted by the automated POSTIDENT procedure used for identity verification, the research data center will consider the case individually and seek an alternative solution for identity verification.
