# Supplementary material for: Data from the ACHILLES (Assessing Daily Changes in Individual Prerequisites of Learning Success) Project
Source: J Open Psychol Data. 2026 Oct 15;14:6. doi: 10.5334/jopd.182 (PMC13674530; doi:10.5334/jopd.182)
Supplement: Supplementary file 1 [file jopd-14-182-pr1.pdf]

Initial Peer Review Round for “Data from the ACHILLES (Assessing Daily Changes in Individual Prerequisites of Learning Success) Project”

Dear Jasmin Breitwieser, Garvin Brod,

Thank you for submitting your manuscript, “Data from the ACHILLES (Assessing Daily Changes in Individual Prerequisites of Learning Success) project”, to Journal of Open Psychology Data.

Following peer review, we have reached a decision regarding your submission. We would like to invite you to revise your manuscript before it can be accepted for publication.

Both reviewers evaluated your data set and the submitted data papers positively and found the contribution valuable. They also provided several constructive suggestions that could strengthen the manuscript. The full reviewer reports are included at the bottom of this email. Rather than reiterating all comments, I would like to highlight a few key points that should be addressed in your revision:

First, Reviewer 1 requested clarification of the data access procedure, including whether researchers outside Germany are also eligible to request access. Please describe potential access restrictions in more detail.

Second, please provide a description of the 20 indicators derived from the log data, preferably in a table (see Page 22).

Third, please include a table that provides an overview of the files available in the data repository, together with a brief description of the contents of each file (see also Reviewer 2’s corresponding suggestion). As a template, you may wish to consult Table 2 in the following publication: https://doi.org/10.5334/jopd.79. In this context, please also consider Reviewer 1’s suggestion to provide English translations of the file names, as they are currently available only in German.

Fourth, please add the publications based on the described data set (Section 2.8) to the reference list and cite them appropriately within the manuscript.

In addition to these points, please address the remaining comments and suggestions provided by both reviewers or provide a rebuttal if you decide against addressing them.

Instructions for how to resubmit your article online are pasted below. Please ensure that your revised files adhere to our author guidelines, and that the files are fully proofed prior to upload. Please also include a revised version of your article with 'tracked changes',

adding comments where appropriate, to indicate the revisions made, in addition to a brief document outlining how you have responded to the reviewers' requests.

If you have trouble processing the revisions, our Help Center (https://help.u-community.io) or downloadable PDF (https://bit.ly/Author-Guide-OJS-3) may be able to help. If not, please get in touch and we'll be happy to help.

Please also ensure that all copyright permissions have been attained for any figures/tables you have included.

Please could you have the revisions submitted not later than September, 21th 2026. If you cannot make this deadline, please let us know as early as possible.

Kind regards,

Timo Gnambs

If external review was obtained, the comments from the reviewers are below:

Reviewer 1:
Recommendation: Revisions Required

Comments to the author(s)

Thank you for the opportunity to review the manuscript, "Data from the ACHILLES (Assessing Daily Changes in Individual Prerequisites of Learning Success) Project." This is an interesting and potentially valuable dataset containing intensive longitudinal data from up to 365 German medical students during their preparation for the second state examination. The dataset is rich and well documented, including the online reports, and the authors clearly outline several forms of potential reuse.

Comments below are grouped by topic.

Data access and governance
• The manuscript states that the dataset is governed by FDZ Bildung's Terms of Use. Based on the information provided on the FDZ Bildung website, the repository appears to offer different levels of access. Please briefly explain why this dataset requires the protected-access procedure involving POSTIDENT verification.
• Relatedly, the POSTIDENT procedure appears to accept only certain identity documents. Please clarify whether this might affect access for researchers outside Germany, whether

researchers worldwide are eligible to access the dataset, and whether alternative identity-verification procedures would be available if an applicant's documents are not accepted by Deutsche Post.

- The authors state that contractual agreements prevent sharing the original platform data and that only 20 derived variables are provided. Please clarify whether the cited examples of prior data use relied on these derived variables or on the original, non-shareable data. If they relied on the original data, the manuscript should clearly state that these analyses may not be reproducible or extendable using the deposited dataset alone.

Data organisation and usability

- The ZIP archive contains a large number of data files, particularly log files. Please consider adding a table listing each file or file type, briefly describing it, and, for log files, explaining the naming convention. Such a table could also provide English descriptions alongside the German file names. This information could be added to Table 1 or presented in a separate file-level overview.
- On my system, extracting the archive was estimated to take approximately 1 hour and 25 minutes and involved 10,776 items. Given that the individual log files appear to be very small, please consider providing them in a more consolidated format, for example, as a single combined dataset or a smaller number of grouped files. This could substantially improve the usability of the deposited data.
- The authors state that "variable names and some categorical value labels are provided in German." This wording may suggest that English-language value labels are not available, even though they appear to be included in the English versions of the Method Reports. Please clarify this point. If possible, consider providing English labels (and file names) by default; otherwise, revise the sentence to make clear that English-language labels are available in the accompanying reports.
- Table 1: The Method Report also appears to include the number of variables in each domain. This was helpful for assessing the relative breadth of coverage across areas, including potential limitations in domains such as demographics. Please consider adding this information to the table.

Design

- The manuscript should explicitly define the target population when introducing the study design and sampling procedure. Although medical students appear to be the population of interest, the manuscript also uses the broader term "students," which may create ambiguity. Please distinguish the target population from the study population and the convenience sample, or state explicitly if no inference beyond the study population is intended. This would make it easier to assess the scope of inference and the limitations of the sampling design.

Manuscript structure and reporting

- The citation to Winne (2005) appears insufficient to support the claim that self-regulated learning has been studied over the past five decades, as a 2005 source cannot cover developments during the subsequent two decades.

• In the sentence “The primary learning activity, strongly supported by the digital learning platform AMBOSS,” please consider deleting “strongly.” The term seems evaluative and may read as an endorsement of the private provider unless it is supported by evidence or defined more precisely.
• Please spell out REDCap at first mention and provide the acronym in parentheses.
• In the sentence “Third, after studying, students evaluate their performance in relation to their goal and derive conclusion for future adjustments to their learning approach,” the word “conclusion” should probably be plural.
• The background section is somewhat lengthy and at times includes details that may be better placed in the “Materials/Survey Instruments” section. Please review the section for material that could be moved or removed, particularly where the same information is repeated later in the manuscript.

Reviewer 2:
Recommendation: Revisions Required

Comments to the author(s)

This manuscript describes a valuable and well-documented dataset with considerable potential for secondary analyses in the field of self-regulated learning. The manuscript is well written, clearly structured, and provides a comprehensive description of the study design, the collected data, and their potential for reuse.
I particularly appreciated the transparent reporting of the study design, including the explicit description of the unintended extension of the ambulatory assessment period due to the REDCap configuration error. Such openness increases confidence in the quality and transparency of the dataset. I also found the accompanying methodology report to be exceptionally comprehensive. The detailed documentation of the datasets, variables, coding schemes, and derived indicators greatly facilitates data reuse and will be highly valuable for future researchers. Furthermore, I appreciate the authors' balanced discussion of the dataset's limitations, including the use of single-item measures, the limited within-person reliability of the working memory assessment, and the absence of raw log data. These limitations are presented transparently and provide important guidance for researchers intending to reuse the dataset.
Overall, I believe this dataset represents a valuable contribution to the field and that the manuscript requires only minor revisions before publication.
Minor:
- Page 5: The citation (Ku & Ho, 2010, 2010) appears to contain a duplicated publication year. Please check and correct the reference if necessary.
- Page 6: In the sentence "...students reappraisal or suppression of emotions...", the possessive apostrophe is missing. It should read "students' reappraisal or suppression of emotions".
- Reuse potential / Limitations: The limitations section introduces the discussion of the working memory assessment with "Regarding the measurement of working memory performance...". Since this represents a separate limitation from the previous paragraph,

consider introducing it as "Second," for consistency with the subsequent numbering ("Third," and "Finally,").

- Repository structure: While the accompanying methodology report provides excellent documentation of the variables and datasets, a brief overview table of the repository structure (e.g., listing each dataset, its level of observation, and the linking variable) would further facilitate navigation for secondary users.
- Reuse potential (Suggestion): The Reuse Potential section is informative and well written. However, it could be made more concise by reducing the number of illustrative examples of potential future research questions while retaining the main strengths and applications of the dataset.
